# Loot

**Published:** 1868-10-15

**Authors:** 


### *LOOT.*

---

### *Abortion as a Cause of Insanity.*

The Superintendent of the Michigan Insane Asylum, in his report just published, says: "Mental derangement has generally occurred as a result of local injury, and the serious impairment of general health, directly traceable to the criminal act. In a few cases it has operated as a moral cause; as, for instance, when the unfortunate sufferer has borne a child which has been permitted to remain with her only long enough to show the unhappy mother the priceless value of the gift she had previously refused to accept. In these cases the immediate cause of the insanity is remorse. Unless this most disastrous practice be speedily arrested by the efforts now being used to suppress it, and by more stringent laws, severely punishing all parties implicated, it will materially increase the number of female patients annually presented for treatment."—*N. Y. Medical Journal.*

### *Antiseptic Properties of the Sulphites.*

Recent experiments have shown that the sulphites of lime, hyposulphite of magnesia, and the sulphites of magnesia and soda, possess all the antiseptic properties of sulphurous acid, with the advantage that their action is more uniform and certain. In experimenting on animals and on himself, Dr. Polli (*Med. Times and Gazette*) found that large doses could be taken without risk. On killing animals treated with sulphites, and others not so treated, he found that the former were more slow to decompose, and, indeed, remained quite fresh when the others had become putrescent and offensive. Another series of experiments showed that the administration of the sulphites was sufficient to effect a more or less rapid cure where blood-poisoning was present, as in fevers.—*Humboldt Medical Archives.*

### *Prolapsus Ani.*

Dr. Schartz, in *Hufeland's Journal*, recommends for this affliction a solution of the *ext.* of *Nux vomica*, of the strength of one or two grains to the ounce of distilled water. Of this solution he gives six to ten drops every four hours. This is the dose for very small children; to larger children fifteen drops at the same intervals. Children at the breast, two or three drops.—*Nashville Journal of Medicine and Surgery.*

### *Styptic Paper.*

The universally used perchloride of iron is too well known to need notice, but we see that it has been assuming a new and perhaps more convenient form. A French inventor has prepared styptic paper so that one can carry it in his pocket. He takes a carefully tinned vessel, and dissolves one pound gum benzoin and one pound of rock alum in four gallons of water. The paper is dipped into this, and when dry a solution of the perchloride of iron is applied with a roller or brush, and the paper is then kept in a dry place. It must certainly be valuable for small wounds. Is it of use in dentistry? We think so. When we hear more of it, we will report upon its value. We are ambitious to make this a dental newspaper.—*Dental Office and Laboratory.*

### *Carbolic Acid and Creasote.*

As we receive many letters asking questions in reference to these substances, we have thought that a few words in reference to these hydro-carbons, which are so much used in dentistry, might not be inappropriate to the occasion. It is a common error to suppose that creasote and carbolic acid mean the same thing. They have many properties in common, and their chemical influence upon other bodies is often analogous, yet, as they are derived from different sources, and the quantative dissimilarity of their elements, it is evident that they are not the same. They are of comparatively recent discovery, and are already recognized as of high value in medicine and in the arts.

Creasote was discovered in 1830 by Richenback, and is prepared from wood tar, which is distilled till it has the thickness of pitch, and then suffered to stand till two layers are formed, the lower one only of which contains the creasote, and after it goes through a series of changes by the introduction of modifying chemical agents, the pure creasote of commerce is produced. Pure creasote is nearly colorless, is of an oily nature, and at a temperature of 380° can be entirely volatilized without leaving any residuum. It has a burning, caustic taste, and a smoky, penetrating odor, and is inflammable. When applied to the skin, it is highly caustic. Its use is evidently that it coagulates the albumen when used for dental purposes. It is irritant, escharotic, and styptic, when locally used, and is regarded as a general antiseptic and narcotic. In certain diseases of the throat it makes a valuable gargle.

When it is designed to detect the difference between carbolic acid and creasote, it may be done by noting the boiling point, creasote boiling at 307°, and carbolic acid at 368°. The sesqui-chloride of iron will also make a blue color when added to the carbolic acid, but does not affect the creasote.

Carbolic acid is called *phenyl*, *phenic acid*, etc. As its name indicates, it is an acid derived from carbon oil. It is usually prepared from coal oil, and is produced in the form of colorless, deliquescent crystals, which fuse at 95°, and have the odor of smoke, and an acrid taste. It is a powerful antidote to the formation of putrefaction in animal tissues, and has been used for preserving dead bodies, etc. In the dissecting room it is much used of late to preserve bodies while under the surgeon's knife. A French author says that eight grains in solution is enough for this purpose. It is taken inwardly, in doses of one or two drops, to prevent vomiting, and for other complaints. It is a powerful styptic, and when applied to a bleeding surface it checks the hæmorrhage at once. When applied to purulent or other offensive discharges, it is of great value. When applied to an exposed pulp, it obtunds the sensibility. A pledget of cotton dipped in a drop of the acid, and allowed to remain a few minutes, relieves the pain much better than creasote. The mouth-wash used by dentists is made by putting five drops of carbolic acid in a tumbler of water.

Carbolic acid and glycerine is much used for sloughing ulcers, and for various ulcerations. In time, we believe that dentists will find it invaluable. No office will be without it pure, or in some of its combinations.—*Ibid.*

### *Minute Investigation of the Kidney.*

M. Rendonsky (*Virchow's Archiv.*, bd. 41, 1867) gives the following results of his investigations of the minute structure of the kidney: 1st. The uriniferous tubules are continued into the capsules of the malpighian bodies, or terminate in blind extremities. 2nd. The malpighian capsules are placed on convoluted tubules, lined by nucleated epithelium; other and smaller canals, supplied with transparent ephithelium, communicate finally with these tubuli. 3rd. Straight tubuli are connected with some capsules, which, at a short distance from these capsules, show the characters of the convoluted tubuli. 4th. The convoluted and the straight tubes are connected by tubuli, which are lined by transparent (non-nucleated) epithelium; the convoluted tubes are in communication with the capsules, and the straight tubes open into the pelvis of the kidney. 5th. Henle has described canals with transparent epithelium, as continuations of the tubuli uriniferi, which are really blood-vessels.

### *Local Treatment of Soft Chancre.*

The application of carbolic acid causes the rapid destruction of the ulcerating surface, with decomposition of the poison, and without any considerable degree of pain. The surface of the sore is turned white by the acid; this becomes a thin, dry, yellow scab, which separates in about two or three days. The application should be repeated to the third or fourth time, when it may be found that the sore has healed under the scab. The healing of the sore is generally completed in an average of ten to fourteen days.—*British Med. Journal.*

### *An Aged Primipara.*

In response to the inquiry made through the London *Lancet*, with respect to child-bearing in advanced life, Dr. Cachot, of St. Mary's Hospital, informs us that he delivered in that institution a female of her first child, at the age of 53 years, and again in sixteen months. The labor in both confinements was tedious, from inertia of the uterus, and required the forceps. The mammary glands enlarged, but produced no milk. The children lived in both cases.—*Pacific Medical and Surgical Journal.*

### *Veratrum Viride in Constipation.*

In an obstinate case of habitual constipation, Dr. T. C. Miller (*Journal of Materia Medica*) gave three drops, five times a day, of the *tr.* of *Veratrum viride*, and in the course of two weeks effected an entire recovery.—*Record.*

### *The Siamese Twins.*

Prof. Paul F. Eve, in the *Richmond and Louisville Journal*, re-asserts the opinion of many physicians and surgeons who examined the case a score or more years since, that the connection between the Siamese Twins is mainly a cartilaginous one, (probably a prolongation of the ensiform cartilages), and that their rumored intention to soon visit Europe for disunion purposes, is a needless expense, as any surgical tyro, even, could as well separate them by the knife or *ecraseur*.

### *Carbolate of Quinia.*

Prof. Wenzel remarks (*Jahrbucher der Gesammten Med.*, Aug. 28, 1868), that carbolic acid, which in solution acts as a poison upon the lower animal organisms, is borne in proportionate, though large doses, by the higher animals and man, when introduced into the body in a diluted state. It was administered to some animals with advantage in their food in England at the time of the rinderpest. With bases, even weak ones, such as quinia, carbolic acid loses in a great degree its irritating properties at the point where it is applied; when combined in the proportion of two equivalents of the acid to one of quinia, the compound is characterized by a slight sharpness, and a decidedly bitter taste. Prof Bernatzik proposes a preparation composed in this manner, and he hopes that it will prove an energetic disinfectant for internal use. G. Braun has given it with benefit in puerperal diseases, and Duchek in several typhous cases, and in one of pyæmia. Pills containing 1 grain of quinia with 6-10ths of a grain of carbolic acid were given repeatedly without causing the slightest inconvenience, and according to these statements 3 to 6 grains of carbolic acid were given daily without injury. The compound was prepared by dissolving 60 parts of carbolic acid with 100 of quinia, in 300 of highly rectified spirit, filtering the solution, distilling and evaporating to the consistency of turpentine, and then mixing some extract of acorus and powdered cassia.—*Amer. Jour. Med. Sci.*

### *Aphonia of Nearly Two Years' Duration Cured by Electrical Stimulation of the Inferior Laryngeal Nerve. By Dr. Philippeaux.*

Various methods of treatment had been unsuccessfully tried in a case of aphonia which had originated two years previously, and which was supposed to be due to paralysis of the nerves of the vocal cord. The patient was a healthy female, twenty years of age, It was ultimately decided by Dr. Philippeaux to try the effect of electrical stimulation, applied in such a way as to directly influence the inferior laryngeal nerves. For this purpose, one metallic pole was inserted into the lower and posterior portion of the pharynx, and the second was placed on the skin over the crico-thyroid muscles. A current of considerable strength was passed between these two points: almost immediately after the closure of this current the patient started, uttered a loud cry, and began to speak with a facility equal to that which she had possessed before the commencement of the aphonia. Dr. Philippeaux remarks that he has frequently met with success in treating aphonia by electricity, but never before had he the good fortune to obtain so instantaneous and perfect a cure.—*Edinburgh Medical Journal.*

### *Vesicular Bronchitis of Both Lungs in an Infant —Recovery. By David Wooster, M.D.*

S. S——, a female child aged eighteen months, had measles of a very severe type. The throat was sore and the tonsils had ash-colored spots. On the subsidence of the eruption an oppressive, dry cough supervened, and the tongue became dry and brown. The pulse rose to 160 during the accession of fever, and fell to 80 when the fever remitted; it seldom remained above 100 long at a time. The child had the usual remedies prescribed in similar cases; had Trousseau's mixture, gum syrup and ipecac (the antimony omitted in consequence of vital depression); the diachylon jacket was also applied next the skin.

I was unable to visit my little patient at night, in consequence of my own poor health at the time. An excellent physician who was called in the night for three nights, intelligently supplemented my treatment by giving syrup of senega, nitre for scanty urinary secretion, and small doses of calomel for inactive bowels. Several other things were done, besides the infinite items of domestic treatment for infants, such as onion syrup, flaxseed poultices, mustard cataplasms, etc.

On the fifth morning after the bronchitis became marked, I called at 10 A.M., and found the child in clonic spasms, with pulse exceedingly weak and filamentary, double strabismus and dilated pupils. Soap and-water enemas relieved the spasm, but the pulse remained small, feeble and intermittent, and less than 100. I now asked the parents if they would trust me implicitly and solely with the child, living or dying. They consented to obey my directions implicitly and in silence, until the child should convalesce or expire.

I ordered the tables to be cleared, all damp clothing and poultices and cloths to be removed from the child, the room to be cleared of guests and friends, placed a thermometer in a convenient place and directed the temperature of the room to be maintained at 70° night and day, while air should be admitted from an open window; made a toddy of one part whisky and six of sugar and water, of which a teaspoonful should be given every hour while the pulse should remain below 110. Directed beef juice to be given in such proportion as to amount to six ounces in twenty-four hours, milk as much as the child should wish. No other medication or treatment was permitted.

The child began to mend directly, and although exceedingly feeble for some days, at the expiration of less than two weeks it was entirely well.

This was a typical case of vesicular bronchitis supervening when the system was already in a deteriorated condition in consequence of a severe exanthema; and a case which would have doubtless terminated fatally had we persisted in the ordinary medical treatment.—*Pacific Journal.*

### *Vaginismus.*

Scanzoni objects (*Monatschrift fur Gabertskunde*, *Detroit Review*) to the severe operative measures adopted and recommended by Dr. Marion Sims for this troublesome difficulty. He says: "Of more than a hundred cases that have fallen under his observation, in times past, he has been completely successful in the treatment of all to which he was able to give his personal attention, without in a single case having recourse to the knife. The first condition of success is complete sexual abstinence; for the first three or four days a tepid sitz bath should be used night and morning; warm local bathing with aq. Goulardi, or the same applied with lint, several times a day. Defecation must be regulated, and friction from motion carefully avoided. After a few days the sensibility of the parts will be so much allayed that a solution of *Arg. nitrat.* x.–xx. grs. to ℥i. may be applied with a brush. After about eight days continuance of this treatment, vaginal suppositories of *Ext. belladonna* and Cacao butter may be placed behind the hymen and in contact with it, daily. These remedies, either alternately or simultaneously, must be continued until every trace of inflammation has disappeared, and the normal sensibility is restored. Generally two or three weeks will be required to attain these objects. Then dilatation must be commenced, but for this purpose sponge tents are useless. A

graduated series of milk-glass conical specula are best adapted to this object. After the first slightly painful attempt, the patient generally will be able to introduce it with facility, and it may be allowed to remain from one-half to one hour. Even when the hymen remains, it will not be necessary to incise it, as dilatation can be effected without recourse to that measure. At first the dilator may be used every two or three days, then every day or twice a day for two or three hours, gradually increasing the size of the dilator until the object shall have been attained, which in some instances may require an instrument admitting dilatation, as that of Segalas. Sitz-baths, *Belladonna*, and pencilling with *Nitrate of silver* may be required from time to time, and the cure will usually be completed in from six to eight weeks. It will be seen that, although the treatment of Sims is attended with an equally satisfactory result, it is of a much more serious character (a fatal result from hæmorrhage is reported to have occurred) than the treatment adopted by Scanzoni; and after the operation the success of the treatment depends generally upon the subsequent dilatation. The time required, moreover, is nearly the same by either process. Hence the procedure of Sims is in no respect to be regarded as an advance in gynecology, as little so, even, as is his operation of splitting the cervix uteri, which, with such glowing eloquence, he lauds for the cure of dysmenorrhœa and sterility. Scanzoni is thoroughly convinced that these are but surgical splurges, that impose on the inexperienced only, while the professional expert justly ignores them. He predicts their fate. For a time they will be the theme of much remark; soon after they will pass into merited oblivion."

### *Cataract.*

We have long believed that more thorough efforts should be made by physicians to remove, or rather prevent, the full formation of cataract. Twenty years ago, some cases were reported by Dr. James Bryan, of Philadelphia, then President of the College of Physicians and Surgeons, of that city, in which cataract was stopped in its progress by medical treatment. We have had two such cases lately, and they have disappeared under the treatment very similar to that then used, and we believe many cases of incipient cataract may be thus removed. The effort is surely worth the trial.—*Guardian of Health.*

***Caustic Ligature.***

M. Vallette, of Lyons, recommends strongly that in treatment of erectile tumors by the multiple ligature, a seton, impregnated with *Chloride of zinc*, be passed through the strangulated mass. Fifteen or twenty hours afterward, he cuts down on the subcutaneous caustic seton, and removes it, introducing a further quantity of caustic, if necessary. He says that this method is much less painful than the simple ligature, and more efficacious than it, or than the treatment by caustics only.—*Reporter*.

***Sterility.***

W. C. Amussat, Jr., reports a case of sterility in a male cured by an operation for phimosis. One year afterwards, his wife gave birth to a son.

***Epilepsy.***

W. M. Cornell, M.D., LL.D., in the *Guardian of Health*, gives the following "handful of recipes" brought to him by an epileptic patient. They had been given him by a medical gentleman of eminence, who was for many years at the head of one of the hospitals in Massachusetts:

℞ 1. Nitrate of silver half a drachm, extract of stramonium one scruple, nux vomica half a drachm; made into fifty pills with crumbs of bread.

℞ 2. Strychnia twelve grains, vinegar two ounces. Mix, and take ten drops three times a day in sugar and water.

℞ 3. Wild cherry and prickly-ash bark, equal parts, eight ounces; put it in a gallon of soft water; boil till two-thirds are evaporated; add two pounds of brown sugar. Dose, a wine-glassful before each meal.

℞ 4. Chloric ether, two ounces, spirits of camphor two drachms; sweet spirits of nitre two ounces; mix. Dose, a teaspoonful three times a day, in water.

℞ 5. Oxide of silver one drachm, extract of conium two drachms, colocynth one drachm; mix, make into sixty pills. Dose, two three times a day.

℞ 6. Tincture of nux vomica four ounces, tincture of stramonium one and a half ounces, chloric ether two and a half ounces; mix. Dose, a teaspoonful three times a day,

℞ 7. Fowler's solution half an ounce, chloric ether two ounces; mix. Dose, fifty drops at bed time, in water.

℞ 8. Extract nux vomica two scruples, extract stramonium half a scruple, oxide of silver one drachm; crumbs of bread sufficient to make sixty pills. One three times a day.

℞ 9. Tincture of nux vomica three ounces, compound spirits of lavender two drachms; mix. Dose, twenty-five drops three times a day, in sugar and water.

It seems scarcely necessary to add that the treatment was totally unavailing. It is probable that in most cases of epilepsy the difficulty is mainly one of habit. Any strong mental influence may at times control its manifestation. Hence the long list of so-called remedies, which have really no influence save from the patient's faith. Physiological and and pathological *habits* need more attention than they have yet received. This not in epilepsy only.

### *Foreign Intelligence.*

*Action of Mercury.*— At the recent meeting of the British Medical Association, in Oxford, Professor Hughes Bennett read an abstract of the results which had been arrived at by the Edinburgh Committee. The Committee, after a laborious investigation on the action of mercurials on dogs, arrived at the conclusion, that, whether administered in large or small doses, the preparations of mercury exert no cholagogue action upon that animal — in fact, that they always diminish the flow of bile. How far this report can serve to throw light npon the action of mercurials on man, is, however, a matter upon which more than one opinion can be held. In the course of their investigations, the Committee have found that mercurials, when administered in large doses to dogs, purge them; and, when in smaller and frequently repeated doses, induce the same group of phenomena, which are observed in men under the same circumstances, viz., fetor of the breath, salivation, and ulcerations of the gums. Having accurately ascertained these facts, the Committee appear to consider that the fact that mercurials fail to increase the flow of bile in the dog, affords an almost positive proof that these drugs do not exert a cholagogue action in the case of man. The experiments supported also the modern view that the diversion of the bile through a fistulous opening out of the body does not materially interfere with the intestinal functions, but leads to exhaustion of the body altogether. Dr. B. W. Richardson accepted the report as a model of scjentific work, but urged still that mercury did exert a beneficial effect. and that experience confirmed its value. Was it possible, he asked, that mercury acted on the pancreatic gland as it did on the salivary glands, and that it caused an increase of pancreatic secretion? Dr. Bennett, in reply, said it was quite possible the pancreatic function was modified under the action of mercury, for, as one of the tables indicated, the pancreas in five cases was reported as very vascular.—*Medical News.*

### *Ovariotomy.*

Dr. Dunlap, of Springfield, Ohio, has performed ovariotomy on 38 patients, since 1843. Of these, 13 were unmarried.

The operations were all by the long incision, and only two were without anæsthetics. Nine died after operation; one from peritonitis, two from hæmorrhage, one from chloroform, one from accidental overdose of morphine, one complicated with cancer, one from exhaustion, one from congestion of the brain, and the ninth from excessive vomiting. Three of the successful cases have died since their recovery from the operation, of other diseases; the remainder are all now living, and in good health.—*Boston Medical Journal.*

### *Remittent Fever in Rome.*

M. Pantaleoni, of Rome, says that the remittent fever which prevails in that city, is found in two distinct forms. 1st, the gastric, which is mild and easily managed; 2nd, the nervous form, which is ataxic, but different from typhoid in many of its characteristics, as the absence of abdominal symptoms, pain, diarrhœa, etc., and of the rose spots, as well as the anatomical lesions discoverable in typhoid after death. The French soldiers, during their stay in Rome, had typhoid fever the first six months, and after that would contract nerous fever.— *Medical Record.*

### *Thymic Acid.*

This acid, obtained from the essential oil of thyme, has been proposed as a succedaneum of carbolic acid or creasote. It emits no disagreeable smell, and is powerfully antiseptic. Its composition is $C_{20}\ H_{14}\ O_{2}$. In a concentrated form it may take the place of nitrate of silver; and, as an antiseptic, it should be disolved in 1,000 parts of water, with the addition of a little alcohol.—*Ibid.*

